# Bacterial Endocarditis Caused by *Sphingomonas paucimobilis*: A Case Report and Literature Review

**DOI:** 10.1155/2020/7185834

**Published:** 2020-10-09

**Authors:** Kara Rognrud, Andrew M. Diaz, Collin Hill, Melissa A. Kershaw

**Affiliations:** ^1^Department of Cardiology, Christian Hospital, St. Louis 63136, MO, USA; ^2^Department of Medical Education, A.T. Still University, Kirksville 63501, MO, USA

## Abstract

A 47-year-old male with no significant medical history was hospitalized for bacteremia and diagnosed with endocarditis. The organism isolated was a Gram-negative bacillus—*Sphingomonas paucimobilis*. There are only a few reported cases of endocarditis caused by *S. paucimobilis*, and to our knowledge, this is the first in the United States.

## 1. Introduction

Currently in the *Sphingomonas sensu stricto* genus, there are more than 30 species, and of those, only *S. paucimobilis* has clinical significance [1]. It was originally believed that *S. paucimobilis* was not a major pathogen. With more investigation, it appears it should not be overlooked as a possible cause of nosocomial infections and could warrant consideration for inclusion in standard screenings for infectious pathogens [2, 3]. Multiple case reports conclude that it is mostly contracted in the hospital or community setting [4]. We report a case of *S. paucimobilis* endocarditis in a patient with no comorbidities or previous medical history.

*S. paucimobilis* is ubiquitously found in the environment, specifically in soil and water. The bacterium has been isolated from nearly every source of water, including sterile water used in hospitals. Additionally, the bacterium has been shown to form biofilm in pipelines that carry drinking water [5]. Many infections caused by the bacterium are nosocomial and are associated with indwelling medical devices, such as catheters and central venous lines [6]. Furthermore, a majority of infections occur in patients with significant comorbidities. Malignancy, end-stage renal disease, diabetes mellitus, and use of immunosuppressive drugs are the most common risk factors [4]. The patient presented in this case report had no comorbidities but did work on refrigerators and cooling systems, which could have been a reservoir for the pathogen.

## 2. Case Report

D. S. is a 47-year-old Caucasian male who arrived to the emergency department reporting a four-month history of intermittent fever and chills lasting a few days at a time, lightheadedness, fatigue, and tachycardia. He denied a recent history of illness or rash and denied any allergies. Personal past medical history and family history were unremarkable. He worked as a supervisor for a refrigeration plant and had no drug or tobacco use. Temperature upon admission was 39.6°C. Initial labs and imaging revealed a white blood cell count of 12.2 K/cumm (4–12 K/cumm), hematocrit of 32% (40–52%), normal platelets, normal renal function, negative urinalysis, negative chest X-ray, and a negative influenza test. Blood cultures were obtained, and he was provided IV hydration and one 500 mg dose of IV levofloxacin. He was subsequently discharged with a 10-day course of once daily 500 mg PO levofloxacin and instructed to follow-up with his primary care provider.

A few days later, at his follow-up appointment, his levofloxacin was discontinued as his PCP was unsure of what infection was being treated by the antibiotics. Blood cultures from the ED grew *S. paucimobilis* on one of two samples, and repeat blood cultures were obtained. The repeat blood culture showed no growth. A transthoracic echocardiogram (TTE) was recommended for possible bacteremia.

**Figure 1:**
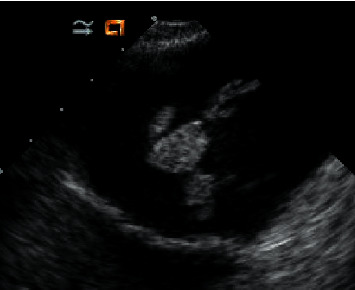
TEE image showing a 3.6 cm pedunculated mass within the left atrium.

**Figure 2:**
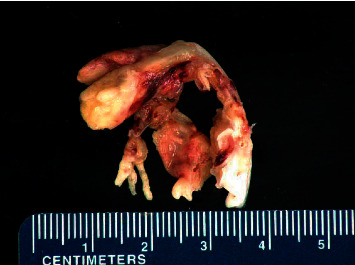
Gross pathology of the mitral valve with a left atrial polypoid mass measuring 4.0 cm × 1.5 cm.

Ten days after the initial presentation, the patient arrived at an outside medical facility with continued symptoms and new right lower extremity edema and pain. Generally, he was a healthy appearing Caucasian male in no acute distress. Review of systems was remarkable for lightheadedness, most notably when febrile. D. S. denied chest pain, palpitations, dizziness, recent kidney or bladder infections, lung disease, rash, or recent weight change. Vitals included a BP of 138/82, pulse of 68, and a temperature of 36.8°C. Cardiac exam showed regular rate and rhythm with a grade 2/6 apical murmur present with no gallop. The rest of the physical exam, including dentition, was normal. A TTE showed a large, mobile, echogenic structure in the left atrium. A right lower extremity Doppler showed a clot in the right posterior tibial vein. The patient was admitted to the hospital to obtain further evaluation with a transesophageal echocardiogram (TEE). He was started on piperacillin/tazobactam 3.375 g IV every 6 hours and vancomycin 1 g every 12 hours. CT scan of the abdomen and pelvis was performed. A TEE showed a highly mobile 3.6 cm pedunculated mass within the left atrium (Figure 1), thickening of the mitral valve with presence of small mobile masses consistent with vegetations, and moderate mitral regurgitation. He was sent to surgery for mitral valve replacement using a #31St. Jude mechanical valve. The left atrial mass was excised. A tissue culture was negative. Gross pathology showed a left atrial polypoid mass measuring 4.0 cm × 1.5 cm and posterior and anterior mitral leaflets with a pink-tan appearance resembling a valve, with areas of shaggy red-tan tissue resembling vegetation (Figure 2). The pathology report of the microscopic specimen noted the mitral valve leaflets demonstrated fibrous valvular tissue with myxoid degeneration, areas of fibrin with early macrophage organization, dystrophic calcification, and mixed acute and chronic inflammation, consistent with vegetation formation. Infectious disease was consulted, and his antibiotic regimen was changed to meropenem 1 g IV every 8 hours and vancomycin 1 g IV every 8 hours. He was ultimately discharged with plans to complete a 6-week course of meropenem 1 g every 8 hours.

One week after surgery and thirty days from initial presentation, D. S. was seen by the cardiologist for follow-up. He was receiving his IV antibiotics through a PICC line, and at that time, he was asymptomatic with only mild postoperative chest discomfort. He was on Coumadin for his mechanical valve and brief postoperative atrial fibrillation. His three-month follow-up was unremarkable, and the patient was asymptomatic at that time. Upon review of history, it appeared that the source of the infection was likely occupational in origin. D. S. had been informed that, he had been exposed to nosocomial-associated contaminated water or contaminated ventilator temperature probes at his workplace. He is currently doing well, without further complications.

## 3. Discussion

*Pseudomonas paucimobilis* was first isolated in 1977 and characterized as a yellow pigmented, Gram-negative, rod-shaped bacterium with a single flagellum [2]. The bacterium was later reclassified and renamed in 1990 to *Sphingomonas paucimobilis* due to the presence of sphingoglycolipids in the cell membrane [7]. While it is generally considered to be of low pathogenicity due to a lack of lipopolysaccharide A in its cell wall, infections and fatal cases have been reported [8]. The most common reported manifestation of *S. paucimobilis* infection is bacteremia, but other infections have been reported including peritonitis, pneumonia, urinary tract infections, osteomyelitis, splenic abscesses, empyemas, and meningitis [3].

The patient in our case report had an initial blood culture that grew *S. paucimobilis*. However, a subsequent blood culture resulted in no growth. According to the modified Duke criteria, a diagnosis of endocarditis can be made if two major criteria, one minor criterion and three minor, or five minor criteria are fulfilled [9]. In our patient, one major criterion could be fulfilled for evidence of endocardial involvement, and minor criteria could be fulfilled for the presence of a fever and positive blood culture that does not meet major criteria. Although the patient's clinical presentation was classified as possible endocarditis according to modified Duke's criteria, pathology showing active endocarditis confirmed the diagnosis.

In one literature review of published case reports of *S. paucimobilis* infections, the most effective antibiotics for treating *S. paucimobilis* were ampicillin/sulbactam, carbapenem, levofloxacin, piperacillin/tazobactam, and ciprofloxacin [4]. Due to the production of beta-lactamase by the bacteria, *S. paucimobilis* is typically resistant to penicillins and first-generation cephalosporins [8]. Our patient was initially treated with levofloxacin but was ultimately treated for *S. paucimobilis* endocarditis with IV meropenem for 6 weeks.

A review of the current literature shows three rare cases of endocarditis caused by *S. paucimobilis*. The three cases were found in Mexico, China, and Taiwan. A recent retrospective case series on the epidemiology and prognosis of infective endocarditis in China analyzed 154 patients who had endocarditis. In this study, the etiology of one patient's endocarditis was listed *S. paucimobilis* [10]. Similarly, a second retrospective analysis focused on the clinical features and mortality of 87 patients with endocarditis in Central Taiwan which showed one case of endocarditis caused by *S. paucimobilis* [11]. Lastly, a case report published in Medicina Interna de Mexico reported a case of *S. paucimobilis* [12]. To date, there have been no published cases of this bacterium causing endocarditis in the United States. Important information, such as patient risk factors, treatment regimen, and outcome of the patients, is omitted in the cases found in China and Taiwan. The patient case from Mexico stated the patient improved after a two-week course of antibiotics but the antibiotic of choice was not specified.

## 4. Conclusion

While infections caused by *S. paucimobilis* are still uncommon, the number of reported cases has been increasing in recent years. To our knowledge, we have reported the first case of *S. paucimobilis* endocarditis in the United States. This patient was immunocompetent with no comorbid conditions and no known risk factors for either endocarditis or infection with *S. paucimobilis*. This patient shows that *S. paucimobilis* is of increasing importance and should be considered as a possible etiology of endocarditis, even in healthy patients.
